# A Study for Determining the Inclination of the Occlusal Plane from the Mandibular Functional Trajectory

**DOI:** 10.1155/2022/6713881

**Published:** 2022-07-01

**Authors:** Kazuhiro Ikeda, Shuichiro Yamashita

**Affiliations:** Department of Removable Partial Prosthodontics, Tokyo Dental College, Tokyo, Japan

## Abstract

**Background:**

During the fabrication of prostheses, the putative occlusal plane is generally determined based on morphological criteria such as Camper's plane. There is a possibility that morphological indexes deviate from their original positions over time. The current study investigated whether functional indexes could be used to determine occlusal planes instead of morphological indexes.

**Objectives:**

The objectives of the present study were to investigate the relationship between the maxillary occlusal plane and mandibular functional trajectory and to consider a method for reconstructing a lost occlusal plane based on functional trajectories.

**Methods:**

Thirteen healthy dentulous individuals were recruited for the study. Using a jaw motion tracking device with 6-degrees of freedom, the trajectories of the mandibular first molar and incisor during masticatory movement or tapping movements were measured.

**Results:**

The closing angle of the mandibular first molar to the maxillary occlusal plane was converged and averaged 74.1° just below the intercuspal position, which is the terminal position of masticatory movement. This angle was positively correlated with the closing angle of the incisal point during tapping movement. The regression equation obtained was *y* = 0.37*x* + 45.99, where *x* was the closing angle of tapping movement and *y* was the closing angle of masticatory movement.

**Conclusions:**

It was suggested that when determining lost occlusal plane, the occlusal plane can be derived using the closing angle of the incisal point during tapping movement.

## 1. Introduction

Improvements in oral health services in recent years have led to a global increase in the number of elderly individuals with more than 20 remaining teeth [1], but problems arise when appropriate prosthetic treatment is not performed in partially edentulous regions. One example is an inappropriate occlusal plane resulting from movement of adjacent teeth and extrusion of opposing teeth. An incorrect occlusal plane increases the difficulty of fabricating a prosthesis. Therefore, early prosthetic intervention is crucial to maintain the occlusal plane in order to facilitate the creation of a suitable prosthesis.

During the fabrication of prostheses the putative occlusal plane is generally determined based on morphological criteria such as Camper's plane. Camper's plane is widely thought to be almost parallel to the occlusal plane in normal dentition [2–7], but some studies have indicated otherwise [8–12]. This discrepancy between reports is most likely due to the fact that the occlusal planes were established using a morphological index. There are differences in individual features in the maxillofacial region and aging causes changes over time such as bone resorption and muscle weakening. Furthermore, there are clear individual differences in the process of aging. In conjunction with such changes, there is a possibility that morphological indexes deviate from their original positions over time. The current study investigated whether functional indexes could be used to determine occlusal planes instead of morphological indexes.

One study investigating the occlusal plane from a functional perspective indicated that mandibular morphology and tooth loss affects the inclination of the occlusal plane [13]. Reports also indicate that there is a correlation between the inclination of the occlusal plane and the angle between the trajectory of masticatory movement on the sagittal plane [14, 15]. In those studies, the trajectory of masticatory movement on the sagittal plane was measured at the incisal point, but the functional center of actual masticatory movement was reportedly the central pit on the mandibular first molar [16]. Studies have also indicated that the movement of the mandibular incisal point during mastication differs from that of the first molars [17]. Many relevant factors thus remain unclarified, such as what the most efficient angle for first molars to come into contact with the occlusal plane during mastication is, and whether the functional trajectory of the first molars corresponds to the occlusal plane when it is set parallel to Camper's plane.

One of the primary objectives of the current study was to investigate the relationship between the maxillary occlusal plane in normal dentition when it was regarded as an occlusal plane depending on a functional index, and the trajectory of the mandibular first molar during masticatory movement or the trajectory of the mandibular incisors during tapping movement. The other was to consider a method for reconstructing a lost occlusal plane based on functional trajectories.

## 2. Materials and Methods

### 2.1. Subjects

The study included 13 healthy dentulous individuals, 10 men and 3 women, and their ages ranged from 25 to 32 years (mean 27.4 years). The inclusion criteria for subjects were as follows: (1) having healthy natural dentition with no history of orthodontic treatment; (2) angle Class I occlusions; (3) no temporomandibular disorders; and (4) not currently undergoing dental treatment. The study protocol was approved by the Ethics Committee of Tokyo Dental College (#0730), and informed consent was acquired from all subjects. Because this was a pilot study, the number of subjects was set to be small.

### 2.2. Measurement of the Mandibular Movement

Mandibular movement was measured using a jaw motion tracking device with 6 degrees of freedom (MM-J2, Shofu Inc., Kyoto, Japan). The respective root mean square errors in the measurement of position and jaw motion were 0.2 mm and 0.1–0.8 mm. A pair of metal clutches were attached to the labial surfaces of maxillary and mandibular incisors to position the sensors. A rapid-cure adhesive agent (Aron Alpha, Toagosei Co., Ltd., Tokyo, Japan) was used to afﬁx the clutch.

Specialized software was developed to analyze mandibular movement. The sampling rate for the measurement of mandibular movement was set at 200 Hz. In the current study, the maxillary occlusal plane—*i.e.*, the incisal edge of the maxillary central incisor to the distobuccal cusp on bilateral maxillary first molars—was used as the reference plane. The *x* axis, *y* axis, and *z* axis, respectively, corresponded to the anteroposterior, horizontal, and vertical directions.

### 2.3. Test Movements

Test movements included masticatory and tapping movements in the natural head position. Each movement was repeated for 20 cycles. Both types of movements were performed in a comfortable sitting position without the head fixed.

### 2.4. Masticatory Movement

Gum (Greengum, Lotte Co., Ltd., Tokyo, Japan) was used to test masticatory movement. After sufficiently softening the gum in the oral cavity, masticatory movement was conducted on the habitual masticatory side. A metronome was used to maintain a rhythm of 1 cycle at 1 Hz.

### 2.5. Tapping Movement

Tapping movement was defined as repetitive opening and closing at the intercuspal position, at a mouth opening distance of 2–4 mm on the habitual closing path. A metronome was used to maintain a rhythm of 1 cycle at 2 Hz.

### 2.6. Relationships between the Morphological Reference Plane and the Maxillary Occlusal Plane

**Figure 1:**
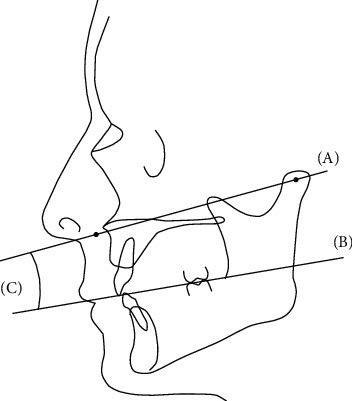
The morphological reference plane and the maxillary occlusal plane. (a) Morphological reference plane; the bilateral arbitrary hinge positions and the inferior margin of the nasal wing. (b) Maxillary occlusal plane; the incisal edge of the maxillary central incisor to the distobuccal cusp on bilateral maxillary first molars. (c) The angle formed by the morphological reference plane and the maxillary occlusal plane.

The morphological reference plane was established using three points; the bilateral arbitrary hinge positions and the inferior margin of the nasal wing. The arbitrary hinge position was set on the line connecting the tragus and lateral canthus, 13 mm in front of the tragus. The angle formed on the sagittal plane by the morphological reference plane and the maxillary occlusal plane was calculated, and the relationships between the planes were analyzed (Figure 1).

### 2.7. Closing Angle

**Figure 2:**
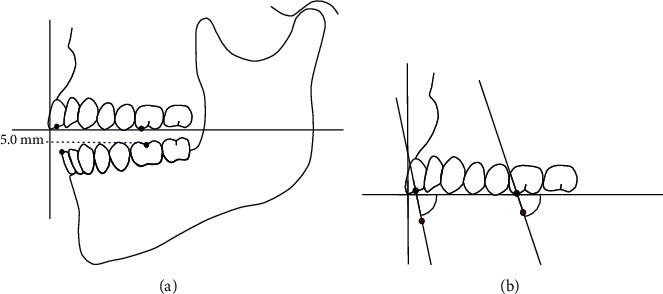
The closing angle during masticatory movement and tapping movement. (a) Six points were set on the trajectory of masticatory jaw movement, 5.0 mm, 4.0 mm, 3.0 mm, 2.0 mm, 1.0 mm, and 0.5 mm. Four points were set on the trajectory of tapping jaw movement, 2.0 mm, 1.5 mm, 1.0 mm, and 0.5 mm. (b) Closing angle at the 5.0-mm point.

The incident angle during mouth closing on the test movement path with respect to the maxillary occlusal plane was investigated in accordance with a study by Ogawa et al. (Figure 2) [15].

### 2.8. Closing Angle during Masticatory Movement

The mouth closing path of mastication was determined on the sagittal plane using the central pit on the mandibular first molar as an indicator. On this path, the angle formed by the straight line that connected the intercuspal position and six positions under the intercuspal position and the maxillary occlusal plane was calculated. This angle was established as the closing angle during masticatory movement. The six positions were 5.0, 4.0, 3.0, 2.0, 1.0, and 0.5 mm below the intercuspal position.

### 2.9. Closing Angle during Tapping Movement

The mouth closing path of tapping movement was determined on the sagittal plane using the mandibular incisal point as an indicator. On this path, the angle formed by the straight line that connected the intercuspal position and four positions under the intercuspal position and the maxillary occlusal plane was calculated. This was established as the closing angle during tapping movement. The four positions were 2.0, 1.5, 1.0, and 0.5 mm below the intercuspal position.

### 2.10. Correlation between the Closing Angle of Masticatory and Tapping Movements

In clinical practice, it is difficult to reproduce a specific point during masticatory movements. Tapping movements are easier to record, and the mouth closing angle thus obtained can be used to determine the maxillary occlusal plane. Therefore, in the current study, the correlations between the closing angles of converging points obtained during masticatory and tapping movements were assessed.

### 2.11. Statistical Analysis

Normality tests were performed on the mouth closing angles during masticatory and tapping movements. Pearson's correlational coefficients and a bivariate linear regression analysis were calculated using SPSS software (SPSS Statistics version 25, IBM Japan, Ltd., Tokyo, Japan). The explanatory variable was the closing angle during masticatory movement and the response variable was the closing angle during tapping movement. The significance level was set at 0.05.

## 3. Results

### 3.1. Relationships between the Morphological Reference Plane and the Maxillary Occlusal Plane

From the left sagittal plane view, if the clockwise direction was positive, the angle of the maxillary occlusal plane with respect to the morphological reference plane was 5.1 ± 7.5° (mean ± standard deviation) (Table 1). This suggests that the maxillary occlusal plane tends to be horizontal compared to the morphological reference plane. The maximum value was 19.1° and the minimum value was −5.1°.

### 3.2. Closing Angle during Masticatory Movement

**Figure 3:**
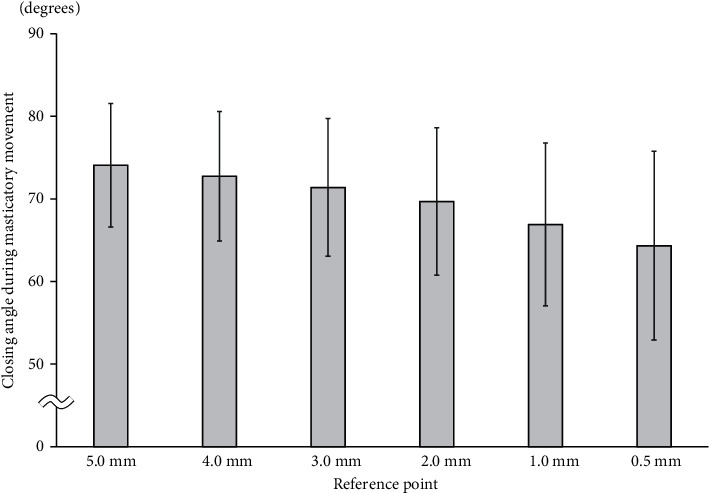
Closing angle during masticatory movement.

During masticatory movement, the angle tended to decrease from 5.0 mm toward the 0.5-mm position. The angle at the 5.0-mm position was 74.1 ± 7.5°, and the standard deviation was smaller than it was at any other position (Figure 3).

### 3.3. Closing Angle during Tapping Movement

**Figure 4:**
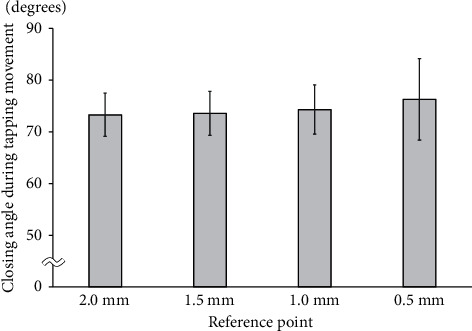
Closing angle during tapping movement.

During tapping movement, the angle tended to increase from 2.0 mm toward the 0.5-mm position. The angle at 2.0 mm was 73.3 ± 4.2°, and the standard deviation was smaller than it was at any other position (Figure 4).

### 3.4. Correlation between Closing Angles during Mastication and Tapping

**Figure 5:**
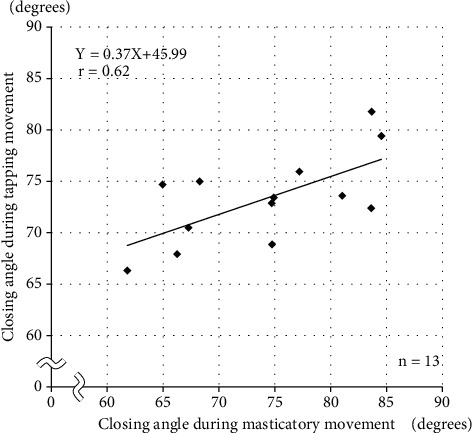
Correlation between closing angles during masticatory movement and tapping movement.

The datasets for both masticatory movement and tapping movement were normally distributed and there was a statistically significant correlation between the closing angles at the 5.0-mm position during masticatory movement and the 2.0-mm position during tapping movement (*r* = 0.62). The *P* value for the test of no correlation was 0.023. The regression equation obtained was *y* = 0.37*x*  + 45.99, where *x* = the closing angle of tapping movement and *y* = the closing angle of masticatory movement (R squared = 0.44, a standard error of the estimate = 3.41). The mean closing angle during masticatory movement was 74.1°, and when this value was substituted into the regression equation the closing angle during tapping movement was 73.4° (Figure 5).

## 4. Discussion

The primary objective of the current study was to determine the occlusal plane from the functional path of movement when reestablishing the occlusal plane after tooth loss. The morphology of the oral cavity changes over time due to functional adaptation. The occlusal plane also changes over time due to mastication; therefore, the path of mastication should be taken into consideration when finding the occlusal plane.

### 4.1. Morphological Reference Plane and Maxillary Occlusal Plane

Camper's plane, hamular notch-incisive papilla plane, or lateral cephalometric radiography can be used as a reference plane when determining the occlusal plane. Among these, the prosthodontic literature suggests that Camper's plane has been the most commonly used. In the present study, Camper's plane was also used as a standard to obtain the morphological reference plane.

In the current study, the mean value was 5.1°, three subjects exhibited values > 10°, and the maximum value was 19.0°. There were a number of individuals whose maxillary occlusal plane and morphological reference plane were inconsistent. The inclination of the occlusal plane is closely related to other occlusal elements; therefore, it is important to determine occlusal planes, via parameters such as functional indicators.

### 4.2. Closing Angle during Masticatory Movement

The current study focused on the closing path of the mandibular first molars during masticatory movement as a functional index of the occlusal plane. This was because the importance of mastication in elderly individuals was a primary consideration. As well as influencing nutritional health, mastication reportedly has a significant effect on dementia [18], thus improving masticatory function general health in the elderly. The first molars can be considered the optimal site when selecting functional indicators because they make a major contribution to the occluding area during mastication [19].

In a previous study, Uesugi et al. [20] reported that there was a significant correlation between the amount of mouth opening and masticatory function. Kitashima et al. [21] reported that food properties are involved in the mandibular position when mouth opening is > 3.0 mm in the masticatory movement path of the mandibular first molars. This information suggested that functional factors were most likely involved at positions a distance away from the intercuspal position in the masticatory movement path; thus, the 5.0-mm point was considered valid.

### 4.3. Closing Angle during Tapping Movement

In current research, a jaw motion tracking device with 6 degrees of freedom is generally used to determine mandibular movement paths. Notably, however, the use of these devices in general clinical practice is very rare. Furthermore, it is impractical to obtain a masticatory movement path at the mandibular first molars, especially at the 5.0-mm position. Therefore, in the present study, tapping movement at the incisal point was used as a functional movement that could be easily obtained. In comparison to masticatory movement, tapping movement at the incisal point is easily obtained; there is little inconsistency between cycles, and the movement can be easily repeated.

In the current study, there was a significant correlation between the closing angle during masticatory movement at the 5.0-mm position and the closing angle during tapping movement at the 2.0-mm position. The following method is suggested for establishing occlusal planes depending on a functional index when fabricating full dentures: (1) Establish a putative occlusal plane based on Camper's plane and obtain occlusal vertical height using the conventional method. (2) Create a gothic arch tracer, set it such that the vertical height is raised 2.0 mm from the acquired height, and set indices such that the incisal edges of the maxillary and mandibular central incisors are clear. (3) Mount the tracer in the oral cavity and direct the patient to perform tapping movement. Measure the angle of the putative occlusal plane and the line connecting the indices of the maxillary and mandibular central incisors where the tapping points converge. (4) Correct the wax rim of the maxilla so that this angle is the closing angle during tapping movement derived from the regression equation (73.4°).

The virtual articulator has gained acceptance in recent years. Its popularity is expected to increase because the articulator can be reproduced on a computer, negating a complicated technical work. Moreover, it is possible to reproduce mandibular movement on the articulator by superimposing mandibular movement data derived from a jaw motion tracking device onto the virtual articulator. If performing such superimpositions becomes more common, the results of the present study can easily be applied.

The main limitation of the current study was that all the subjects were healthy and dentulous. A cephalometric analysis using X-ray images would have allowed a more detailed analysis, but this was not possible due to ethical issues. It is necessary to compare results obtained via the conventional method with those via the methods described herein using edentulous subjects in the future. Despite the limited number, there were no significant gender differences. However, the regression equation of obtaining current study should be scrutinized with subjects of various ages and sexes.

## 5. Conclusion

A study was conducted on healthy dentulous patients to develop a method to determine a lost occlusal plane using a maxillary occlusal plane derived from masticatory movements. The following conclusions were drawn: A study was conducted on healthy dentulous patients to develop a method to determine a lost occlusal plane using a maxillary occlusal plane derived from masticatory movements. The following conclusions were drawn:

1. The closing angle of the mandibular first molars during masticatory movement generally converged at the 5.0-mm position and that of the incisal region during tapping movement generally converged at the 2.0-mm position. There was a significant positive correlation between the two closing angles.
2. When determining a lost occlusal plane, the occlusal plane can be derived using the closing angle at the 2.0-mm position during tapping movement.

## Figures and Tables

**Table 1:** Angle formed by the morphological reference plane and maxillary occlusal plane.

	Mean	Sd	Minimum	Maximum
Angle formed by the morphological reference plane and functional occlusal Plane (degrees)	5.1	7.5	−5, 1	19.0

## Data Availability

The data used to support the findings of this study are restricted by the Ethics Committee of Tokyo Dental College in order to protect subject's privacy. Data are available from author (ikedakazuhiro@tdc.ac.jp), for researchers who meet the criteria for access to confidential data.
